# A Comprehensive Review on Hydrogen Production from Biomass Gasification

**DOI:** 10.3390/molecules31010099

**Published:** 2025-12-25

**Authors:** Mattia Bartoli, Candido Fabrizio Pirri, Sergio Bocchini

**Affiliations:** 1 Center for Sustainable Future Technologies—CSFT@POLITO, Via Livorno 60, 10144 Torino, Italy; fabrizio.pirri@polito.it (C.F.P.); sergio.bocchini@polito.it (S.B.); 2 Consorzio Interuniversitario Nazionale per la Scienza e Tecnologia dei Materiali (INSTM), Via G. Giusti 9, 50121 Florence, Italy; 3 Materials and Processes for Micro and Nano Technologies Research Group (MP4MNT), Department of Applied Science and Technology, Politecnico di Torino, C.so Duca degli Abruzzi 24, 10129 Turin, Italy

**Keywords:** hydrogen, biomass conversion, reactivity, hydrogen economy

## Abstract

Hydrogen production from biomass gasification has emerged as a strategic pathway for achieving carbon-neutral energy systems, circular resource utilization, and sustainable fuel generation. As global energy systems transition toward renewable sources, biomass-derived hydrogen represents a cornerstone of waste valorization, negative-emission bioenergy, and green hydrogen economies. Among all technologies, hydrogen production through gasification is one of the most consolidated routes with plenty of operative industrial-scale plants. The field of gasification is quite complex, and this comprehensive review describes the current scientific and technological achievements of biomass gasification for hydrogen production, describing the effect of feedstock, reactivity phenomena, reactor design, and catalyst systems. Furthermore, we report on a quantitative analysis regarding the operative cost of gasification of biomass compared with green hydrogen production and methane reforming. We provide a complete and synthetic picture for one of the most critical fields in the hydrogen economy that can actively promote a transition towards a more sustainable society.

## 1. Introduction

Energetic transition demands the creation of a technological portfolio able to overcome the actual energetic paradigm based on fossil fuel utilization [1]. The European community has chosen hydrogen-based technologies as a pillar for reaching a net-zero or even a carbon-negative society by the end of 2050 [2]. Hydrogen technologies are rooted in the energetic source and processes used, which has given rise to a color-based classification wherein hydrogen can be white, gray, brown, black, turquoise, yellow, purple, green, or blue [3]. White hydrogen refers to the natural source found in underground deposits and collected during fracking [4]. This source of hydrogen is a non-renewable one, and it is still quite hard to exploit [5]. The more sustainable hydrogen type is the green one [6], produced through an electrolysis feed with renewable energy sources [7,8]. A change in energetic sources leads to yellow and purple hydrogen, in which energy from electric grids [9] or from nuclear plants [10] are used, respectively. Gray, brown, and black hydrogen are produced from oil and coal-derived sources through high-energetic and environmentally unfriendly processes even if they are the more consolidated routes [11,12]. These hydrogen types can be converted to blue hydrogen by coupling them together with decarbonization routes [13]. This type is characterized by a negative carbon footprint due to the associated capture and conversion of the greenhouse gases formed during hydrogen production (i.e., CH_4_, NO_x_, SO_x_, and CO_2_) [14]. As clearly indicated, the blue color refers more to the productive platform rather than to the process used. The last color of hydrogen is turquoise, and it stands for hydrogen produced from thermal treatments of methane [15]. Among the available technologies [16,17,18,19], gasification is the most diffuse and easily implemented on an industrial scale, and the hydrogen it produces is generally classified as biohydrogen [20,21,22,23]. The gasification process is based on a thermochemical oxidative process of biomass in a controlled atmosphere at high temperature, aiming for the production of a syngas of hydrogen streams, and it has been consolidating through the years [24,25,26]. This review provides a comprehensive overview on gasification, highlighting its critical role in an efficient, sustainable, and integrated energetic ecosystem, providing key insights on chemical reactivity, catalysts used, and technologies’ roles.

## 2. Hydrogen Production Through Thermochemical Conversion of Biomass

### 2.1. Hydrogen from Gasification of Biomass: The Chemistry

Biomass gasification is the thermochemical process that converts biomass feedstocks into a solid and gaseous streams through the partial oxidation of biomass at high temperatures under controlled oxidizing atmospheres using a medium such as air [27], oxygen, or steam [28] with or without the use of a catalyst [29]. Contrary to combustion [30], gasification is designed to limit biomass oxidation driving the reactivity towards the production of a gas stream rich not only in CO_2_ but also in carbon monoxide and hydrogen [31].

**Figure 1:**
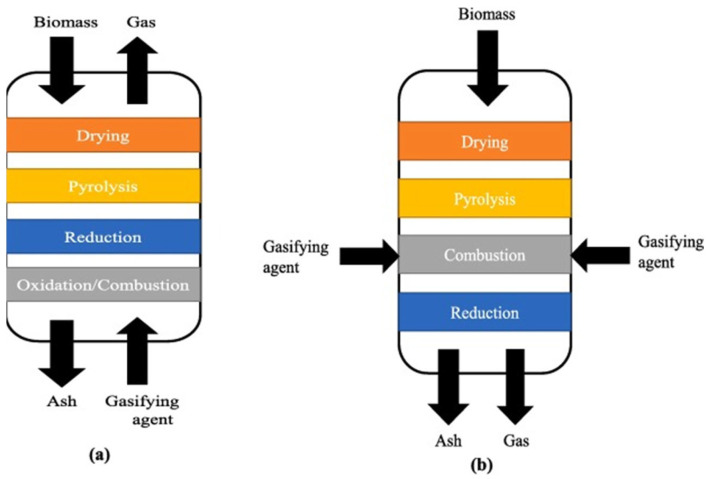
Gasification stages in a (**a**) updraft and (**b**) downdraft gasifier. Picture reprinted from Mishra et al. [33] under CC BY-NC-ND 4.0.

Biomass gasification is composed of multistep processes such as drying, pyrolysis, oxidation, and reduction [32], which can occur in different sections of the reactor as shown in Figure 1 and discussed in the following section.

The four stages of gasification include a complex interplay between different reactions including volatilization of water, thermal cracking of biopolymers, char gasification, tar reforming, and homogeneous gas-phase reactions [34]. The several reaction pathways are characterized by well-defined kinetic, heat, and mass transfer limitations simultaneously affected by temperature [35], biomass composition [36], particle size [37], residual atmosphere (air, steam, CO_2_, O_2_, N_2_) [38], inorganic content [39], use of catalysts [40], and reactor hydrodynamics [41,42].

The first stage is represented by drying, generally occurring below 200 °C, in which the moisture is removed from biomass natural pores and polymer amorphous regions through a combination of evaporation, internal diffusion, and external convection [43]. Biomass drying is a very preliminary step, but it can have a cascade effect on all the other steps, altering heating profiles and particle thermal conductivity [44]. As reported by Hosseini et al. [45], a moisture content exceeding 40 wt.% can induce localized cooling phenomena, suppressing volatile release. The drying rate is related to the biomass structure including the porosity, which retains better and for a longer time the adsorbed water in the fibrous structure [46]. The drying rate is also related to the heating rate and residence time of the feedstock, with fast processes (heating rate over 100 °C/s) being able to induce a brutal disruption of the biomass microstructure known as puffing [47]. By increasing the temperature up to 300–400 °C, pyrolysis begins, and it is characterized by the most complex kinetic and reactive stage as reported in several in-depth studies [48,49,50,51,52,53,54]. The onset and progression of the pyrolytic stage is strongly related to the heating rate, with slow pyrolysis (<10 °C/min) inducing the formation of char and heavy tar through secondary polymerization and condensation [55], while fast pyrolysis (>100 °C/s) promotes depolymerization, volatilization, and production of bio-oil vapors [56]. An ultrafast pyrolysis step (>1000 °C/s) can be achieved by using entrained-flow [57] or plasma-assisted systems [58], and it is able to promote bond-cleavage reactions and fast tar cracking with a massive release of gases, increasing H_2_ formation through enhanced radical chemistry [59]. The complexity of the reaction pathway occurring during pyrolysis includes the degradation of both cellulose and lignin with the formation of anhydrosugars and highly functionalized phenols derivatives, respectively, that evolving into tar, char and gas streams composed of light hydrocarbons and a mixture of H_2_O, CO, CO_2_, and H_2_. The inorganic content plays a critical role in the pyrolytic stage, improving the cracking of the biomass due to the role of species such as potassium, calcium, and magnesium, which are able to lower the pyrolysis activation energies [60]. As reported by Lin et al. [61], silica-rich biomasses, such as rice husk, are characterized by a slower decomposition rate and higher tar yields due to the thermal insulation property of the silica content in their ash. Contrary to drying, residual atmospheres deeply influence pyrolysis chemical pathways [62,63]. As described by Pütün et al. [64], a steam-rich atmosphere promotes mild steam reforming of volatiles, increasing the H_2_/CO ratio, while a CO_2_ atmosphere promotes initial CO_2_–char interactions [65]. Increasing the temperature leads to further heterogenous reactions in both reduction and oxidation stages, listed as follows:

$$\begin{array}{c} C\;+\;H_{2}O\;\to \;\mathrm{CO}\;+\;H_{2}\;(\mathrm{steam–char}\;\mathrm{reaction})\\ C\;+\;{\mathrm{CO}}_{2}\;\to \;2\mathrm{CO}\;(\mathrm{Boudouard}\;\mathrm{reaction})\\ C\;+\;H_{2}\;\to \;{\mathrm{CH}}_{4}\;(\mathrm{hydrogasification})\\ 2C\;+\;O_{2}\;\to \;2\mathrm{CO}\;(\mathrm{partial}\;\mathrm{oxidation})\\ C\;+\;O_{2}\;\to \;{\mathrm{CO}}_{2}\;(\mathrm{oxidation}) \end{array}$$

The first step in the oxidative stage is the char gasification in a temperature range above 700 °C through the steam–char reaction, the Boudouard reaction, hydrogasification reactions, and partial/total oxidation. Steam–char gasification is endothermic and is characterized by a relatively high activation energy [66], while the Boudouard reaction is moderately endothermic and strongly temperature dependent [67]. Hydrogasification is moderately exothermic with slow kinetics [68]; partial/total oxidations are highly exothermic and provide heat for sustaining gasification. These reactions are affected by the microstructure of the char produced during the pyrolytic stage. Chars formed under slow-pyrolysis conditions show ordered aromatic structures and lower reactivity, while ones formed under fast-pyrolysis conditions or high heating rates have more-disordered structures, higher microporosity, and more reactive sites [69]. The production of hydrogen during char gasification is also closely related to the temperature range, and at around 900 °C, the steam–char reaction rates increase sharply together with methane decrement due to the inhibitive equilibrium conditions of hydrogasification [70] and CO conversion through the water–gas shift, shown as follows:

CO + H_2_O → CO_2_ + H_2_ (water shift reaction)

Also inorganic content, especially alkali and alkaline earth metals (K, Na, Ca, Mg) [71], promote steam and CO_2_ gasification by altering the surface functional groups. The catalytic effect of inorganics is particularly significant in agricultural residues that contain high K and Ca [72], while high silica or alumina contents reduce reactivity due to pore-blocking and char encapsulation [73]. The gasification atmosphere composition strongly modifies reaction pathways with steam, which is able to maximize H_2_ production and highly porous char formation [74], while a CO_2_ residual atmosphere enhances the Boudouard reaction with the production of a CO-rich gas [75]. The common use of oxygen or air promotes the formation of heat through exothermic partial/complete oxidation of the feedstock but reduces H_2_ concentration through dilution with N_2_ or through competing exothermic reactions [76]. Interestingly, an increment in the partial pressure of H_2_ reduces the steam gasification through surface hydrogenation reactions and increases methane formation [77]. Furthermore, the use of an operative pressure higher than 30 bar reduces char reactivity by inhibiting gas-phase product buildup and increasing diffusion resistance, while low pressure promotes devolatilization and increases the char pore size [78].

The other process occurring during oxidation is the tar reforming stage, which is crucial for the final syngas purity and hydrogen production. Tar production is undesirable during biomass gasification due to fouling of heat exchangers, causing blocks in filtration systems and poisoning the metal catalysts [79]. Tar reforming is complex, and it is composed of simultaneous thermal cracking; partial oxidation; and steam and dry reforming of condensable hydrocarbons, phenols, furans, cresols, and polycyclic aromatic hydrocarbons [80]. Thermal cracking of tar starts in a temperature range from 800 up to 900 °C when heavy aromatic species undergo dealkylation and radical-driven decomposition to form lighter hydrocarbons and CO, CO_2_, and H_2_ [81]. Nevertheless, thermal cracking can lead to the formation of soot when the residence time, temperature, and radical concentrations are not carefully balanced [82]; this can be mitigated by reforming as summarized below:

Tar + H_2_O → CO + H_2_ (steam reforming of tar) Tar + CO_2_ → 2CO + H_2_ (dry reforming of tar)

Steam reforming of tar is a highly endothermic and strongly temperature-dependent process, with maximum H_2_ yields obtained above ~900 °C, while dry reforming is more relevant in CO_2_-rich atmospheres and contributes to carbon neutrality but requires high energy input [80]. Naturally occurring inorganics can improve both cracking and reforming as shown by the lower tar content observed in high-alkali biomasses such as straws and grasses [83]. The atmosphere significantly influences tar chemistry: a steam atmosphere promotes tar hydrolysis and steam reforming [84], while an oxygen atmosphere reduces tar yield by oxidative phenomena [85]. The use of a CO_2_ atmosphere enhances oxidative cracking through reverse Boudouard reactions [86], while H_2_-rich atmospheres suppress tar cracking due to hydrogenation of radicals [87]. Residence time is also critical, and fast removal of tar vapors decreases secondary polymerization while prolonged residence in high-temperature zones promotes full cracking and reforming [88].

The last stage of gasification of biomass is represented by homogeneous gas-phase reactions that determine the ultimate equilibrium composition of the syngas. These reactions occur between volatile species, radicals, and gases produced during pyrolysis, char gasification, and tar reforming [89], with several reactions summarized as follows:

$$\begin{array}{c} CO\;+\;H_{2}O\;↔\;{\mathrm{CO}}_{2}\;+\;H_{2}\;(\mathrm{methane}\;\mathrm{steam}\;\mathrm{reforming})\\ {\mathrm{CH}}_{4}\;+\;H_{2}O\;↔\;\mathrm{CO}\;+\;3H_{2},\;(\mathrm{methane}\;\mathrm{water}–\mathrm{gas}\;\mathrm{reaction})\\ {\mathrm{CH}}_{4}\;+\;{\mathrm{CO}}_{2}\;↔\;2\mathrm{CO}\;+\;2H_{2}\;(\mathrm{methane}\;\mathrm{dry}\;\mathrm{reforming})\\ \mathrm{CO}\;+\;3H_{2}\;↔\;{\mathrm{CH}}_{4}\;+\;H_{2}O\;\mathrm{and}\;{\mathrm{CO}}_{2}\;+\;4H_{2}\;↔\;{\mathrm{CH}}_{4}\;+\;2H_{2}O\;(\mathrm{methanation}\;\mathrm{of}\;{\mathrm{CO}}_{2}\;\mathrm{and}\;\mathrm{CO}) \end{array}$$

Temperature is the key parameters for regulating this step: high temperatures (>900–1000 °C) are able to promote endothermic steam reforming and dry reforming magnifying H_2_ production and minimizing the methane concentration, while moderate temperatures (400–600 °C) promote exothermic methanation, increasing CH_4_ and reducing H_2_, and low temperatures promote tar condensation and formation of heavier hydrocarbons [89,90]. Pressure influences gas-phase reactions by promoting CH_4_ and CO_2_ formation at high pressures, while H_2_ production is promoted at low pressures [91]. The gas atmosphere also can be used to modulate the kinetic reaction with steam, which enhances the water–gas shift reaction, increasing the H_2_/CO ratio, while O_2_ activates partial oxidation and exothermic heat release [92].

All stage reactivities are influenced by biomass composition in biopolymers (cellulose, lignin, and hemicellulose) and inorganics. Lignin-rich biomass gasification leads to an increase in char and aromatic tar, while cellulose-rich biomass promotes the H_2_ yields at elevated temperatures, and hemicellulose-rich biomass produces more CO_2_ [93].

### 2.2. Hydrogen from Gasification of Biomass: The Catalysts

The performance of catalysts in hydrogen production through biomass gasification is ruled by both intrinsic and extrinsic catalytic activity operational parameters including temperature, steam-to-biomass ratio, gasification atmosphere, pressure, heating rate, catalyst-to-biomass ratio, particle size, residence time, and reactor hydrodynamics [94]. High temperatures (850–900 °C) promote endothermic reforming and maximize hydrogen production reducing tar, but they also increase catalyst sintering and volatilization of alkali metals [95]. Similarly, steam-rich conditions enhance hydrogen yield via steam reforming and the water–gas shift reaction, but they can induce catalyst deactivation through hydroxylation or pore collapse [96], while CO_2_ atmospheres promote dry reforming pathways inducing deactivation through carbon deposition over the catalyst [97]. The presence of sulfur, chlorine, alkali metals, and silica in the feedstock may poison or deactivate catalysts through sulfation, chlorination, sintering, or encapsulation [98,99].

Nevertheless, catalysts are the cornerstone of any efficient biomass gasification process, and they are grouped into four main categories: (i) inherent catalysts already included in the biomass (i.e., alkali and alkaline earth metals) [100], (ii) in-bed catalysts physically mixed with the biomass (i.e., dolomite, olivine, calcite, magnesite, or iron-rich ores) [101], (iii) supported metal catalysts (i.e., Ni [102], Co [103], Fe [104], Rh, Ru, or Mo [105] deposited on structured supports like Al_2_O_3_, SiO_2_, MgO, ZrO_2_, spinels, perovskites, biochar-derived carbons [94]) generally placed in a secondary reactor or reforming section; and (iv) advanced catalytic materials (i.e., metal–organic frameworks (MOFs) [106], perovskites [107]) which have been increasingly investigated for improving stability, tar decomposition efficiency, deactivation resistance, and hydrogen selectivity under industrially relevant conditions [108].

The hydrogen production enhancement promoted by inherent catalysts is firstly exploited at the char–catalyst interface where alkali metals (K, Na) and alkaline-earth metals (Ca, Mg) increase the rates of the steam–carbon and CO_2_–carbon reactions, accelerating char conversion and elevating hydrogen yields [109]. K is able to decrease the activation energy for steam gasification by forming species such as K_2_O, KOH, or K_2_CO_3_, which are able to catalytically activate water molecules, and by facilitating C-O bond formation [110]. Ca catalytic activity enhances steam gasification, mitigating char densification and graphitization during pyrolysis and also preserving porous microstructures that allow faster gasification [111]. Generally, the catalytic role of inherent metals is strongly influenced by biomass species, ash composition, pretreatment, and thermal history [112]. As reported by Adam et al. [113], silica-rich biomasses induce the immobilization of alkali metals via silicate formation, decreasing their catalytic availability and requiring further catalyst addition to achieve hydrogen-oriented syngas compositions.

In-bed catalysts are generally based on minerals such as dolomite [114], magnesite [115], calcined limestone [116], and olivine (Mg_2_SiO_4_/Fe_2_SiO_4_ solid solutions) [117] that promote secondary reforming reactions. Dolomite is among the most used natural catalysts due to its decomposition in CaO and MgO over 800 °C, showing a strong basicity and oxygen-carrying capacity, promoting the catalytic cracking of oxygenated tar compounds, dehydrogenation of heavy hydrocarbons, and in situ steam reforming of evolved volatiles [118]. As reported by Xu et al. [119], CaO is able to boost the water–gas shift reaction, and it can reduce tar formation. Olivine is less active than dolomite, but it shows superior mechanical, attrition, and high-temperature sintering resistance, supporting its use in fluidized-bed gasifiers [120]. As reported by Virginie et al. [121], Fe-doped olivine shows improved catalytic activity due to the formation of FeO_x_, Fe_3_O_4_, and Fe active sites for tar cracking, steam reforming, and deoxygenation promotion, improving resistance against catalyst poisoning by sulfur forming iron sulfides [121].

Supported metal catalysts, especially Ni-containing ones, are among the most effective class of catalytic materials for hydrogen-oriented gasification due to Ni’s activity for C–X (X:C, H, O) bond cleavage, tar reforming, methane steam reforming, dry reforming, and water–gas shift facilitation [102]. Ni/Al_2_O_3_ and Ni/MgAl_2_O_4_ systems have been widely studied due to their high surface area, thermal robustness, and tunable acidity/basicity through modification of the alumina support [122], but Ni catalysts show critical issues including coking, sulfur poisoning, sintering, and volatilization under steam-rich high-temperature environments [123,124,125]. In order to avoid the deactivation, several strategies have been developed for improving Ni catalyst resilience including metal doping (i.e., Ce [126], Zr [127], Mg [128], or Ca [129]) for carbon deposition reduction, use of basic oxides as supports (MgO [130], CaO [131], La_2_O_3_ [132]) to reduce coke formation, and development of Ni-loaded mesoporous catalysts to regulate carbon deposition and improve steam activation kinetics [133].

Fe-containing catalysts represent another important group of gasification catalysts, particularly for tar cracking and water–gas shift enhancement [134]. Furthermore, Fe-containing materials catalyze dehydrogenation and oxygenate reforming pathways and provide redox flexibility (Fe^2+^/Fe^3+^ cycling), which is advantageous in chemical looping gasification in which they act simultaneously as oxygen carriers and as reforming catalysts, enabling autothermal hydrogen generation without nitrogen dilution [135]. Noble metals (i.e., Rh [136], Ru [137], Pd, and Pt [138]) have also proved their exceptional catalytic efficiency for hydrocarbon reforming, tar decomposition, coke resistance, and sulfur tolerance, but their use is limited by high cost.

Recently, biomass-derived carbon catalysts have begun emerging, including activated carbons, hydrochars, and biochar-supported metals that are characterized by high surface area, tunable porosity, and oxygen-containing functional groups that facilitate tar adsorption and reforming [139,140,141]. Also, waste-derived-metal materials such as mud have been widely used due to the combination of low cost and good properties [142]. Advanced materials such MOFs (i.e., ZIF-67 [143], ZIF-8 [144], UiO [145]) have shown a high potential as precursors to highly dispersed metal nanoparticles embedded in carbon matrices with exceptional activity for reforming, high thermal stability after carbonization, and resistance to sintering. Perovskites (i.e., LaNiO_3_ [146], LaFeO_3_ [147], mixed oxide spinels [148]) show tunable oxygen mobility, redox cycling, and high dispersion of catalytically active metals upon reduction, providing stable structures that can withstand harsh gasification environments [149].

Recent developments have used hierarchical [150] and nano catalysts [151] that are able to exploit controlled porosity, surface architecture, and atomic-scale dispersion to enhance steam activation, mitigate sintering, and increase active site density [152].

As summarized in Table 1, the great variety of catalysts available for biomass gasification involve several issues and advantages, and there is no general consensus on which is the most appropriate, and still the choice of the preferred catalyst is related to the particular technology enforced.

### 2.3. Hydrogen from Gasification of Biomass: The Reactors

The design of reactors for biomass gasification is the final stage in which all the consideration about reactivity should be balanced with economics without compromising on hydrogen yield, tar formation, and biomass conversion efficiency. A wide variety of reactor configurations have been developed to face the heterogeneity in composition, reactivity of biomass feedstocks, and outputs. Each reactor is designed to exploit different hydrodynamic characteristics, heat-transfer modes, gas–solid contact patterns, and solved operational challenges [155].

**Figure 2:**
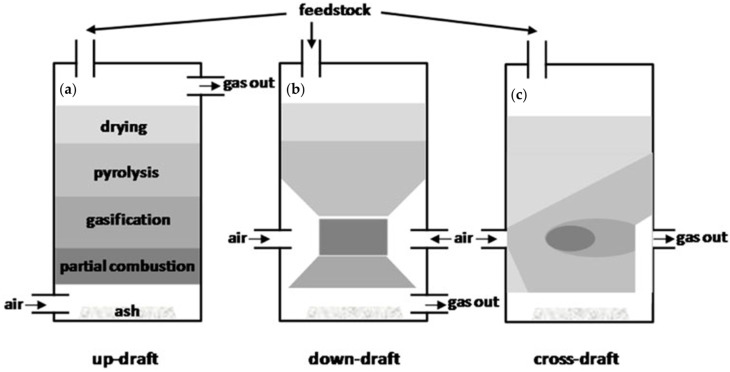
Schemes of (**a**) updraft gasifier, (**b**) downdraft gasifier, (**c**) cross-draft gasifier as reported by Shah et al. [156]. Reprinted with all permissions under CC BY 4.0.

Fixed-bed gasifiers are the oldest and simplest gasification reactors in which biomass falls through the reactor by gravity, while the gasification medium flows either co-current or counter-current. Their advantages include mechanical simplicity, robustness against feedstock variability, and low capital requirements; however, tar production is typically high, and temperature control is limited. Fixed-bed gasifiers are regrouped into downdraft, updraft, and cross-draft gasifiers as reported in Figure 2.

As shown in Figure 2a, biomass and the gasifying agent flow downward in the same direction in downdraft reactor [157]. The gas passes through a high-temperature char bed, which thermally cracks a portion of the tar [158], allowing their use for small-scale or decentralized applications, particularly for syngas feeding internal combustion engines [159]. Downdraft gasifiers achieved moderate yield hydrogen concentrations up to 10–18 vol% depending on steam addition, reactor throat design, and equivalence ratio [160]. As reported by Sandeep et al. [161], the use of steam can significantly enhance H_2_ production through steam–char and water–gas shift reactions in the hot char zone. Nevertheless, this reactor technology shows low throughput due to reliance on gravity-driven flow, has great tar generation when operating below optimal temperature, and is not suitable for high-ash or slagging biomass [162].

As reported in Figure 2b, the updraft gasifiers operate counter-current, and the gasifying medium flows upward, while biomass flows downward. The rising hot syngas dries and pyrolyzes the incoming biomass, transferring heat effectively and achieving high thermal efficiency, but their syngas outputs are lower in H_2_ concentration down to 6–12 vol% than those of downdraft systems, because high tar loads limit downstream catalytic shift or reforming [163]. Furthermore, they accumulate a high tar content, requiring operative conditions for pushing tar cracking or reforming even if they can handle biomasses with a moisture content up to 60 wt.% [164].

The cross-draft systems shown in Figure 2c are characterized by a gasifying medium that flows horizontally through a compact bed with short residence times and high localized temperatures [160]. Cross-draft reactors allow to reach a H_2_ concentration down to 8–15 vol%, but their use is generally limited to low-ash, low-tar feedstocks considering also their low conversion and reduced carbon efficiency.

**Figure 3:**
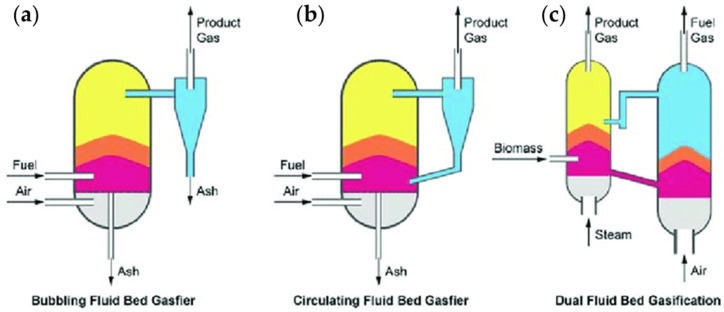
Plant schemes of (**a**) bubbling fluidized bed, (**b**) circulating fluidized bed, and (**c**) dual fluidized bed reactors as reported by Lian et al. [165]. Reprinted with all permissions under CC BY 4.0.

Fluidized bed gasification reactors are among the most widely adopted technologies due to their uniform temperature distribution profile, high heat-transfer rates, and flexibility toward biomass feedstocks with an improved hydrodynamic behavior able to provide superior conversion and lower tar formation compared with fixed-bed systems. Fluidized bed gasification reactors can be classified as bubbling fluidized beds, circulating fluidized beds, or dual fluidized beds as shown in Figure 3.

As shown in Figure 3a, bubbling fluidized bed gasifiers operate with gas velocities slightly above the minimum fluidization velocity for enabling bubbling behavior, enhancing gas–solid contact, and improving devolatilization and steam–char reactions. Bubbling fluidized bed gasifiers can achieve a H_2_ amount up to 15–25 vol% with temperature in the range between 750 and 900° °C, boosting the water–gas shift reaction equilibrium [166]; increasing H_2_ content; and processing several biomasses, including agricultural residues, forestry waste, and energy crops [167]. Nevertheless, they show moderate tar production and can undergo to bed agglomeration when biomasses with a low ash softening temperature are processed [168].

As shown in Figure 3b, circulating fluidized bed reactors operate at higher gas velocities, entraining particles and circulating them through cyclones and return legs, increasing the solids’ residence time and enhancing char conversion [169]. These systems can reach H_2_ concentrations of 20–30 vol% with steam gasification and optimized steam-to-biomass ratios of 0.6, improving the steam reforming of volatiles and tar [170]. The main advantages of circulating fluidized bed reactors are the high throughput and scalability and low tar production compared with bubbling fluidized bed gasifiers, but they require gas–solid separators and detailed control of hydrodynamics. The dual fluidized bed systems reported in Figure 3c are composed of an interconnected steam gasification chamber and an air-fired combustion chamber with bed material circulating between them, transferring heat from the combustion section to the gasification section, thereby enabling indirect gasification without nitrogen dilution [171]. These reactors are actually more efficient, reaching H_2_ concentrations of 35–45 vol% and also associated with the production of CH_4_ and light hydrocarbons, which can be steam-reformed in downstream units to further enrich the H_2_ yield [172]. Furthermore, the outputs are not diluted with nitrogen, and the systems can have a catalytic bed without significant tar accumulation even if their cost, complexity, and deactivation due to the ash accumulation should still be carefully considered [173] to avoid bed agglomeration, which arises from interactions between alkali-rich biomass ash components and silica-containing bed materials [174]. Furthermore, ash–bed material interactions including ash coating, pore blockage, and the modification of the catalytic surface properties of the circulating solids are critical issues to be solved [175] in order to avoid deactivation, altered heat-transfer characteristics, and the increment of attrition rates.

**Figure 4:**
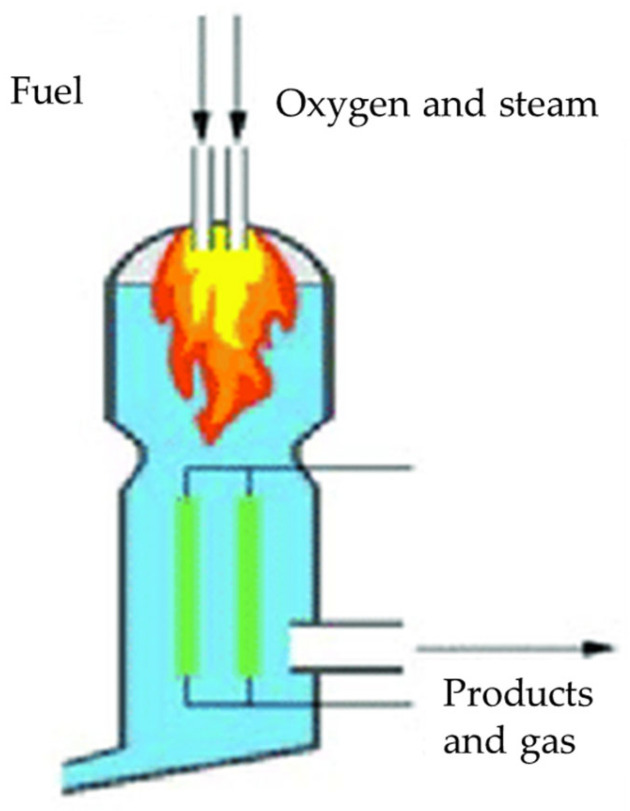
Plant scheme of an entrained-flow reactor as reported by Lian et al. [165]. Reprinted with all permissions under CC BY 4.0.

Entrained flow reactors (Figure 4) operate at high temperatures ranging from 1200 up to 1600 °C with high gas velocities and using feedstocks as very fine powder or slurry-fed to ensure complete gasification in short residence times [59]. This reactor configuration allows the achievement of a syngas with a low tar content and a high H_2_ content up to 50 vol% due to nearly complete conversion of char and hydrocarbons using a high-oxygen gasification agent and increasing operating costs [176].

There are also novel concept of reactors rising in technology readiness including plasma [177], microwave [178], solar [179], and supercritical water reactors [180] that are still far from reaching large industrial scale due to scalability constraints and high cost as summarized in Table 2.

### 2.4. Hydrogen from Gasification of Biomass: Considerations on Economics

The technical and economic landscape of biomass gasification for H_2_ production is shaped by key factors including feedstock cost and availability, gasifier scale, capital investment, catalyst consumption, tar mitigation costs, and purification system requirements. H_2_ produced through biomass gasification is typically characterized by a levelized cost of hydrogen in the range of approximately 2.0–5.0 USD/kg H_2_ that can be further reduced based on the geolocalization of the plant [187]. Capital expenditure for biomass gasification-based hydrogen systems is relatively high, ranging from USD 15 up to 40 per MW as a consequence of the complexity of feedstock handling, gas cleanup, and high-temperature reactors [188]. Nevertheless, operating expenditure is often moderated by the feedstock cost and the low-electricity-intensive step processes [188]. Biomass price is among the largest operational cost component with agricultural residues, forestry waste, and industrial byproducts being the most economically attractive feedstocks due to their wide availability, low cost, and waste reduction [187,189]. Furthermore, there are other disguised costs associated with the biomass such as collection, densification, transport, and storage that introduce significant logistical cost and supply weaknesses [190]. Several studies reported that transportation distance is one of the most sensitive variables affecting the overall production cost, with biomass costs rising sharply beyond a radius of 50 to 75 km from the biomass source [191,192]. A possible approach to mitigate this issue is the decentralization of both biomass processing facilities and gasification plants even if this is not a solution for every scenario. Designing the scale of the gasification plants to match feedstock supply and economic viability is another crucial point, considering that larger plants can benefit from economies of scale, lower per-unit capital costs, and integrated gas cleanup systems with higher efficiency but requiring high- and constant-volume feedstock streams facing the decentralization of many biomass resources [193]. Alternatively, a costly, small modular gasifier can contribute to the creation of a distributed value chain and microgrids with long-term energetic and environmental impacts [194]. All these considerations must be integrated within reference policy frameworks that have a profound influence on the economic feasibility of biomass gasification for H_2_ production. Furthermore, it is critical to paint a picture of the real cost of H_2_ per kg and of yields produced through gasification. As reported by Arena et al. [195], the utilization of a combined of steam gasification and a downstream water–gas shift equilibration allowed a hydrogen conversion of up to 10–14 wt% for each kg of dry biomass with a cost of up to 1.5 USD/kg. Alternatively, steam methane reforming coupled with carbon capture and storage still remains the industrial benchmark of overall process efficiencies of 65–75% depending on the capture rate and integration level [196]. Green hydrogen production through water electrolysis is still the most costly process, with the price driven up by electricity cost and electrolyzer capital expenditure [197]. The actual cost of green hydrogen is close to 7 USD/kg [198], depending primarily on electricity price and capacity factor. Considering electricity prices of USD 40–80 per MW/h [199], capital expenditure of electrolyzers remains close to 1400 USD/kW with an operating expenditure close to USD 40–90 per MW/h [200].

**Figure 5:**
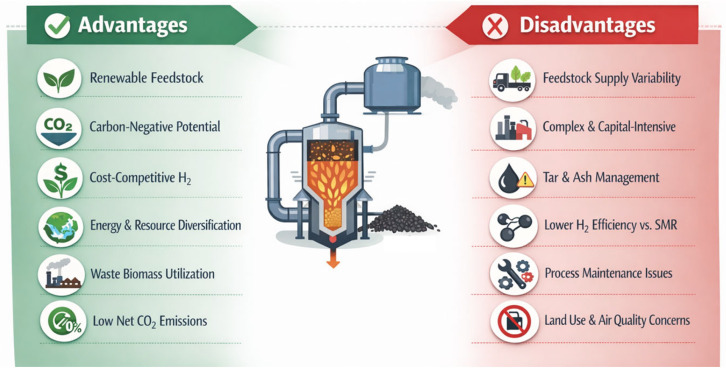
Summary of advantages and disadvantages of biomass gasification.

As summarized in Figure 5, biomass gasification has shown great potential in a hydrogen-based economy due to the competitive cost, solid feedstock gathering, and well-established technology, while the critical points of biomass availability and high operative costs still represent the main drawback of this approach.

## 3. Conclusions

Biomass gasification stands today as one of the most technologically versatile and environmentally friendly routes for producing H_2_ merging renewable energy systems, waste valorization, and low-carbon fuel research and efforts. The combined insights from gasification reactivity, catalytic enhancement, and reactor engineering clearly prove that biomass gasification is not a complicated but a complex scenario, and H_2_ production can be optimized only considering all the parameters at the same time. Some key points clearly emerge from the comparative discussion such as the relevance of the catalyst for modulating the reactivity together with the enforcement of fluidized-bed systems with a particular attention to circulating and dual fluidized beds for reaching an industrial breakthrough. H_2_ production from biomass gasification is actually one of the most balanced technologies between technical viability and environmental friendliness among H_2_ production routes. The continuous advancement in catalyst design, reactor optimization, mechanistic understanding, and system integration will drive the gasification of biomass to a consolidation that provides a significant contribution to energy transition, circular bioeconomy, and a carbon-negative society.

## Figures and Tables

**Table 1:** Comparative performance of main catalyst species for biomass gasification.

Catalyst	Tar Reduction	T (°C)	Coking Resistance	Deactivation Mechanisms	Advantages	Limitations	References
Ni based	High	700–900	Low	▪ Carbon deposition ▪ Sulfur poisoning ▪ Sintering	▪ Very high reforming activity ▪ Strong water–gas shift promotion ▪ Industrial readiness	▪ Rapid deactivation with heavy tars ▪ Sulfur sensitivity ▪ High cost	[102]
Fe based	Moderate	750–950	Moderate	▪ Redox instability ▪ Sintering	▪ Low cost ▪ Sulfur tolerance	▪ Low activity ▪ High temperature demand	[104]
Ca based	Low	650–850	High	▪ Carbonation ▪ Calcination ▪ Sintering	▪ In situ CO_2_ capture ▪ Equilibrium shift toward H_2_ ▪ Very low cost	▪ Limited tar cracking ▪ Cyclic durability	[153]
Dolomite	High	750–900	Moderate	▪ Attrition ▪ Sulfur poisoning ▪ Sintering	▪ Cheap ▪ Dual tar cracking/CO_2_ capture effect	▪ Mechanical fragility ▪ Limited lifetime	[154]
Perovskite oxides	High	700–900	High	▪ Phase segregation ▪ Active site modification	▪ Oxygen mobility ▪ Regenerable ▪ Tunable composition	▪ Poor pilot-scale validation ▪ Complex production	[149]

**Table 2:** Comparison of main gasification reactor technologies.

Reactor Type	H_2_ Yield (vol%) ^a^	Tar Level	Scalability	Limitations	References
Updraft fixed bed	6–12	Very High	Small–Medium	▪ High tar ▪ N_2_ dilution	[181]
Downdraft fixed bed	10–18	Moderate	Small	▪ Limited feed flexibility	[157]
Bubbling fluidized bed	15–25	Moderate	Medium	▪ Bed agglomeration	[182]
Circulating fluidizing bed	20–30	Low	Large	▪ Complex operation	[183]
Entrained flow	45–55	Very Low	Large	▪ High O_2_/pretreatment demand ▪ Fine particle feedstock	[184]
Plasma and microwave	50–60	Very Low	Small	▪ High energy consumption	[185,186]
Supercritical water reactor	40–70	Very Low	Small–Medium	▪ High pressure/corrosion	[180]

(a) Calculated on biomass dry base.

## Data Availability

Data will be provided upon motivated request to the authors.
